# A single-site pilot implementation of a novel trauma training program for prehospital providers in a resource-limited setting

**DOI:** 10.1186/s40814-019-0536-0

**Published:** 2019-12-05

**Authors:** Nee-Kofi Mould-Millman, Julia Dixon, Andrew Lamp, Shaheem de Vries, Brenda Beaty, Lani Finck, Kathryn Colborn, Kubendhren Moodley, Amanda Skenadore, Russell E. Glasgow, Edward P. Havranek, Vikhyat S. Bebarta, Adit A. Ginde

**Affiliations:** 1 University of Colorado, School of Medicine, Aurora, CO USA; 2 Department of Emergency Medicine, University of Colorado, School of Medicine, 12401 E. 17th Ave, B215, Aurora, CO 80045 USA; 3 Western Cape Government Health, Emergency Medical Services, Bellville, Cape Town, South Africa; 4 Adult and Child Consortium for Health Outcomes Research and Delivery Science, University of Colorado, Aurora, CO USA; 5 Department of Biostatistics and Informatics, Colorado School of Public Health, University of Colorado, Aurora, CO USA; 6 College of Emergency Care, Western Cape Government, Bellville, Western Cape South Africa; 7 Department of Medicine, Denver Health Medical Center, Denver, CO USA; 8 Office of the Chief Scientist, 59th Medical Wing, Joint Base San Antonio-Lackland, San Antonio, TX USA

**Keywords:** Pilot study, Feasibility study, Implementation science, Global health, Resource-limited, Education, Effectiveness, Prehospital, Trauma, Emergency medical services

## Abstract

**Background:**

Prehospital (ambulance) care can reduce morbidity and mortality from trauma. Yet, there is a dearth of effective evidence-based interventions and implementation strategies. Emergency Medical Services Traumatic Shock Care (EMS-TruShoC) is a novel bundle of five core evidence-based trauma care interventions. High-Efficiency EMS Training (HEET) is an innovative training and sensitization program conducted during clinical shifts in ambulances. We assess the feasibility of implementing EMS-TruShoC using the HEET strategy, and feasibility of assessing implementation and clinical outcomes. Findings will inform a main trial.

**Methods:**

We conducted a single-site, prospective cohort, multi-methods pilot implementation study in Western Cape EMS system of South Africa. Of the 120 providers at the study site, 12 were trainers and the remaining were eligible learners. Feasibility of implementation was guided by the RE-AIM (reach, effectiveness, adoption, implementation, and maintenance) framework. Feasibility of assessing clinical outcomes was assessed using shock indices and clinical quality of care scores, collected via abstraction of patients’ prehospital trauma charts. Thresholds for progression to a main trial were developed a priori.

**Results:**

The average of all implementation indices was 83% (standard deviation = 10.3). Reach of the HEET program was high, with 84% learners completing at least 75% of training modules. Comparing the proportion of learners attaining perfect scores in post- versus pre-implementation assessments, there was an 8-fold (52% vs. 6%) improvement in knowledge, 3-fold (39% vs. 12%) improvement in skills, and 2-fold (42% vs. 21%) increase in self-efficacy. Clinical outcomes data were successfully calculated—there were clinically significant improvements in shock indices and quality of prehospital trauma care in the post- versus pre-implementation phases. Adoption of HEET was good, evidenced by 83% of facilitator participation in trainings, and 100% of surveyed stakeholders indicating good programmatic fit for their organization. Stakeholders responded that HEET was a sustainable educational solution that aligned well with their organization. Implementation fidelity was very high; 90% of the HEET intervention and 77% of the implementation strategy were delivered as originally planned. Participants provided very positive feedback, and explained that on-the-job timing enhanced their participation. Maintenance was not relevant to assess in this pilot study.

**Conclusions:**

We successfully implemented the EMS-TruShoC educational intervention using the HEET training strategy in a single-site pilot study conducted in a low-resource international setting. All clinical outcomes were successfully calculated. Overall, this pilot study suggests high feasibility of our future, planned experimental trial.

## Background

Trauma is a leading global cause of mortality in persons between 5 and 44 years of age [1]. Each year, there are over 6 million trauma deaths worldwide. Furthermore, injured persons in low-and-middle income countries experience a disproportionately large burden (over 90%) of post-injury death and disability [1–4]. Traumatic injuries and mortality rates globally are expected to continue to rise in incidence, necessitating additional effective strategies to manage this growing global public health crisis [3].

High-quality prehospital (i.e., ambulance-based) care is a critical component of trauma care. Prehospital care can avert 54% of all mortality from emergency conditions, including trauma, in low-and-middle income countries [5]. Despite existence of evidence-based interventions, such as on-scene hemorrhage control and maintaining short scene times, few effective implementation strategies exist to introduce interventions into clinical practice in this setting [6–8].

In low-and-middle income countries, limited resources and strained clinical services often mean traditional educational models (e.g., classroom and simulation training) are difficult to implement and poorly attended, and result in variable outcomes [9–11]. Continuing education can be a cost-effective and sustainable strategy to improve the quality of prehospital trauma care in resource-limited settings [5, 9–11]. Yet, well-described evidence-based interventions and implementation strategies tailored to low-and-middle-income countries are lacking in the scientific literature, with less than 2% of Emergency Medicine guidelines being developed in low-and-middle income countries [11, 12]. More evidence is needed regarding effectiveness of interventions (the “what”) and implementation strategies (the “how”) to impact provider- and patient-level outcomes in prehospital trauma care in resource-limited settings globally [13].

In 2016, we developed a novel educational intervention of bundled trauma care (termed, EMS Traumatic Shock Care [EMS-TruShoC]), which is implemented using a novel training strategy (termed, High-Efficiency EMS Training [HEET]) based on adult-learner principles. In general, bundling of intervention components, usually 3 to 5 items, helps increase clinical uptake [14]. The EMS-TruShoC bundle was developed in 2016, using an international expert panel consensus process to select and assemble existing evidence-based interventions relevant to prehospital trauma care in resource-limited settings [15]. HEET is a novel low-dose, high-frequency, training and sensitization program designed to improve trauma knowledge, attitudes, and skills efficiently during clinical shifts in the back of the ambulance. HEET was modeled after Helping Babies Breathe^TM^, a globally acclaimed training program with 46% mortality reduction in perinatal asphyxia of neonates in resource-limited settings [16].

South Africa was the initial test site for EMS-TruShoC and HEET. South Africa, a middle-income country with high income inequality, has an exceptionally high prevalence of inter-personal violence resulting in 7 times the global mean trauma mortality rate, and loss of 1 million disability adjusted life years (DALYs) in 2000 [2, 17]. The Western Cape Province, approximately 130,000 km^2^ with over 6 million people in 2011, has over 1 million persons estimated to live in dense, informal settlements, where gang warfare and interpersonal violence are major contributors to the burden of trauma [18, 19].

The Western Cape Government Department of Health operates a public emergency medical services (EMS) system that provides prehospital (ambulance) services for the Western Cape [20]. Western Cape EMS transports over 500,000 patients per year, with 40% due to trauma. The system employs about 2000 providers and operates 250 ambulances, distributed over 10 health districts. Western Cape EMS has a well-delineated and distributed management structure, and continuing education is a top organizational priority. In the current state, a quarterly 2-day training program, delivered by experienced EMS educators, is the cornerstone of continuing education in Western Cape EMS. Trainings are conducted at each ambulance base, and providers are encouraged to participate in on their off-days in exchange for educational credits. Success of this program is limited by poor attendance, programmatic reach, and limited training resources.

The overarching purpose of this pilot study is to evaluate the feasibility of implementation and the feasibility of assessing outcomes. If the pilot study satisfies predetermined criteria for success, we will subsequently conduct an experimental trial to gain robust evidence regarding implementation of HEET and clinical effectiveness of EMS-TruShoC.

## Methods

### Objectives

The primary objective is to assess the feasibility of implementing EMS-TruShoC using the HEET strategy. The secondary objective is to evaluate feasibility of assessing implementation and clinical effectiveness outcomes.

### Design

This is a single-site, prospective cohort, pilot study using a multi-method outcomes assessment. The delivery and feasibility assessment of HEET implementation was guided by an implementation science framework, RE-AIM (reach, effectiveness, adoption, implementation, maintenance) [21, 22]. Clinical outcomes were assessed using a previously developed standardized chart abstraction and analysis procedure [23, 24].

### Logic model

**Fig. 1:**
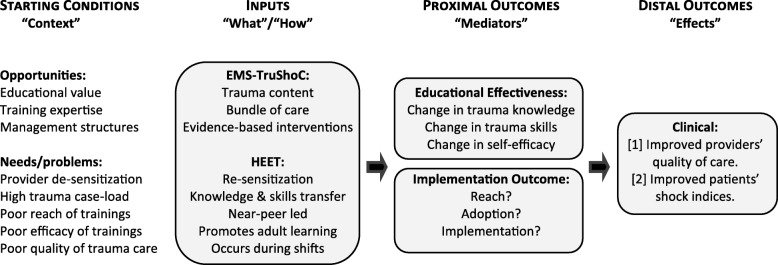
Logic model illustrating how HEET and EMS-TruShoC may impact clinical outcomes

The logic model underpinning this implementation science project is congruent with PRECEDE-PROCEED, a widely used model in public health for bringing change in behavior. The model suggests that behavior change is influenced by both individual and environmental factors [25]. We posit that effective implementation of EMS-TruShoC, using the HEET strategy, should improve providers’ trauma knowledge-attitudes-skills, thereby translating to improvements in their clinical quality of trauma care, and ultimately improving patients’ clinical outcomes (Fig. 1).

### Setting

The pilot was conducted at one busy ambulance base, called “Northern Division,” within the Cape Town metropolitan area. The Northern Division base operates 13–15 ambulances daily, each staffed by a pair of providers, in 12-h shifts beginning at 07:00 and 19:00. In 2017, providers at the Northern Division transported about 7000 trauma patients, with 39% due to gunshot wounds and 43% from assaults and transport related injuries [26]. Traumatic shock comprises 6–7% of total annual trauma volume.

### Participants

Northern Division has about 120 providers at the ranks of basic, intermediate, and advanced life support (BLS, ILS, ALS, respectively). All received international standard training in trauma care in their foundational education. Staff attrition is about 5% per year, and providers generally feel burned out and frustrated by the strained trauma care system [27]. Burnout and de-sensitization contribute to poor quality of trauma care [27]. In the HEET program, all ALS and ILS providers are eligible to be trainers, and all non-trainers are learners. Additionally, we also included EMS charts of patients greater than 17 years of age treated by providers in the study. Since the study focus is traumatic hemorrhage, we excluded injuries with the following mechanisms: drownings, hangings, poisonings, toxic ingestions, burns, bites or stings.

### Intervention components

EMS-TruShoC is a bundle of 5 core, evidence-based, trauma interventions (derived from a prior international expert panel consensus process). The EMS-TruShoC core bundle includes the following: (i) on-scene hemorrhage control, (ii) scene time less than 10 min, (iii) large bore intravenous catheter insertion, (iv) high-flow oxygen administration, and (v) transport to a trauma center [15]. Each component is standard of care and achievable in Western Cape EMS with existing resources.

### Implementation

HEET consists of structured trainings, divided into 8 sessions or modules, which were delivered by peer trainers (called facilitators) in 15-min sessions occurring every other week, during shifts, and in the back of ambulances. A small subset of all available ambulances were reserved for training, and training time was protected from being dispatched into service. Using peer facilitators in a 2:1 learner-to-facilitator ratio, HEET promotes learning intimacy and relationship building. Furthermore, training is conducted within the patient compartment of the ambulance so as to better approximate clinical context and environment while minimizing operational impact. Content is intentionally repeated to encourage retention. For example, the components of the core bundle are defined in each training session. Training sessions have a deliberate focus on the three domains of learning: knowledge transfer, skills practice, and self-efficacy enhancement. Assessments similarly focus on all three domains. Training modules use common clinical case scenarios to promote relevance and enhance adult-learning. In this pilot, HEET facilitators were ILS or ALS providers who elected to participate, and were specially trained in a 2-day program. A Western Cape EMS-appointed HEET Steering Committee oversaw the program. The Committee was comprised of six senior educators and two quality improvement managers—each with over 15 years of experience in Western Cape EMS. A subset of this committee with most familiarity of Northern Division (the “HEET Team”) conducted the pilot implementation.

### Outcomes

Feasibility of implementation was the primary outcome, and feasibility of assessing implementation and clinical outcomes data were secondary outcome. We assessed implementation outcomes using the RE-AIM framework [21, 22, 28] (except for maintenance dimension which was not relevant to this initial shorter term pilot) and intervention outcomes (clinical effectiveness) using shock indices and clinical quality of care [29, 30].

### Sampling

We planned to enroll all EMS providers working clinical shifts during the pilot study since the future study will pragmatically enroll all providers at participating ambulance bases; hence, no sample size estimates were calculated for this pilot study. The HEET Team solicited 12 volunteer facilitators through word of mouth to all ALS and ILS providers. Participation requirement of facilitators was simply a keen interest and being available at work during planned implementation months. Benefits of facilitation, emphasized by the HEET Team, included gaining educational credits, satisfying maintenance of certification requirements, and relationship-building with learners. Although facilitators volunteered, all remaining BLS and ILS staff on duty at the Northern Division were required to participate as learners since this was an on-the-job activity.

### Procedures and data collection

Facilitators were trained by the HEET Team in September, 2017. Implementation was conducted at the Northern Division from October to December, 2017. There were 5 sources for quantitative data:

(i) Learner assessments—assessed knowledge, attitudes, and skills using verbal case vignettes, observation, and written questionnaires, respectively; collected pre- and post-training
(ii) Training rosters—collected names, training dates/times; completed by shift managers after each training session
(iii) Training evaluations—provided anonymous feedback on quality of content, satisfaction, relevance, and length of training; confidentially completed by learners and facilitators after each training session
(iv) Exit survey—assessed overall program experience, satisfaction, problems, willingness to adopt; completed by learners, facilitators, and base managers upon completion of program
(v) EMS clinical charts—retrospectively abstracted by a research associate using a validated chart abstraction tool; charts were completed by Northern Division providers during routine trauma care

Investigators (NM, JD) and trained research associates collected qualitative data upon program completion. We conducted interviews with learners, facilitators, base managers, and the HEET Steering Committee regarding their experiences with the program. Interviews were one-on-one, private, and semi-structured. We took detailed notes during interviews, then transcribed notes into a Microsoft Word (version 10, Redmond, WA) document. All data collectors were blinded to clinical outcome.

### Analysis

Overall criteria for evaluating success (feasibility) of the pilot study were defined as follows: (i) continue to main study without modifications, i.e., feasible as-is; (ii) continue with modifications and/or close monitoring; (iii) stop—main study not feasible [31].

RE-AIM provided a framework to collect and organize implementation data. Quantitative and qualitative data were collected for 4 of the 5 RE-AIM dimension: *R*each, *E*fficacy, *A*doption, and *I*mplementation Fidelity. *M*aintenance, defined as the existence of the institutionalized program after 6 months, was not applicable to this pilot study (as trainings lasted 10 weeks), and therefore not assessed. Each RE-AIM dimension contained one or more indices (Table 1). Data quality, difficulty of collecting data, and missing data were also tracked and documented. RE-AIM data was used to evaluate both implementation feasibility (the primary outcome) and implementation data collection (a secondary outcome). Feasibility of implementation was assessed by averaging 11 key feasibility indices within the RE-AIM framework (Table 1)—clinical and educational effectiveness data are not key feasibility indices, so excluded. The average score for the primary outcome was defined a priori, via deliberation and consensus decision among the investigators, as:

- ○ 80–100% is excellent implementation feasibility,
- ○ 60–79.9% is good implementation feasibility,
- ○ 40–59.9% is fair implementation feasibility, and
- ○ < 40% is poor implementation feasibility.

**Table 1:** Results applied to the RE-AIM framework

Dimension	Quantitative measures					
	Index	Data	Data quality	Difficulty of collection	Missing data	Key feasibility index
Reach (Did we reach the target audience?)	Participating learners (%)^	92/109 = 84%	+++	+	Rarely	Yes
Effectiveness (Individual level outcomes resulting from the program.)	Change in providers’ knowledge scores (post − pre = difference, %)	52% − 6% = 46%	++	++	Rarely	-
	Change in providers’ skills scores (post − pre = difference, %)	39% − 12% = 27%	++	++	Rarely	-
	Change in providers’ self-efficacy ratings (post − pre = difference, %)	42% − 21% = 21%	+	++	Rarely	-
	Improved quality noted in 5 item core bundle of care (%)^$^	3/5 = 60%	+	+++	Often	Yes
	Clinically improved patients’ shock indices (%)	38% − 28% = 10%	+	+++	Occasional	-
	Learner evaluations with satisfaction score ≥ 7 out of 10 (%)	485/526 = 92%	+++	++	Rarely	Yes
	Facilitator evaluations with mean satisfaction scores ≥ 7 out of 10	145/156 = 93%	+++	++	Rarely	Yes
Adoption (Indications that stakeholders and users within the institution will adopt this program.)	Participating facilitators (%)	10/12 = 83%	+++	+	Rarely	Yes
	Facilitators surveyed who would participate again (%)	5/6 = 83%	++	+	None	Yes
	EMS leaders surveyed responding HEET is a good fit for WCG EMS (%)	8/8 = 100%	+++	+	None	Yes
Implementation fidelity (Did we implement the program as we intended?)	Proportion of content delivered as originally planned (%)*	90%	++	+	None	Yes
	Number of training modules delivered as originally planned (%)*	6/8 = 75%	++	+	Rarely	Yes
	Proportion of trainings starting with 15 min of intended (%)	127/164 = 77%	++	+	Occasionally	Yes
	Proportion of sessions (trainings & evals) lasting 15 min or less (%)	131/164 = 80%	++	+	Occasionally	Yes
Average of key implementation feasibility indices (SD)=		83% (SD = 10.3)				
Average of all RE-AIM implementation indices (SD) =		68% (SD = 27.7)				

*K-A-S* knowledge-attitudes-self-efficacy, *WCG* Western Cape Government, *SD* standard deviation

^Learner participation defined by those completing ≥ 75% of training modules. ^$^Learner and facilitator patient charts were included in clinical outcomes analyses. *Index that was subjectively determined through conversation with implementation team. ^+^Compare mean return rate of forms in first 2 modules versus last 2 weeks of implementation

Data quality: + is low; ++ is average; +++ is high. Difficulty of data collection: + is easy; ++ is moderate; +++ is difficult

The feasibility of assessing implementation effectiveness (secondary outcome) was assessed by appraising the completeness of qualitative and quantitative data within the RE-AIM framework. The presence of data in most (defined as ≥ 80%) indices would favorably suggest progression to main study without any modifications, whereas presence of data in some (40–79%) indices would suggest modest modifications needed, while the presence of data in only a few (< 40%) indices would suggest very poor feasibility of implementation outcomes data collection in the future trial.

Qualitative data were thematically analyzed, by reviewing interview notes, to provide participant and stakeholder experiences. This was not a formal mixed-method study as there was no a priori plan to integrate nor co-analyze quantitative with qualitative data. Considering the pilot nature of this study, a formal deductive and thematic analysis, using independent reviewers and consensus discussion, was not conducted.

Feasibility of calculating clinical outcomes in the future trial was assessed in our pilot study by the success of calculating the average change in patients’ shock index, and separately, the average change in providers’ quality of traumatic shock care score. Shock index is heart rate divided by systolic blood pressure (calculated at the scene and on hospital arrival) [29]. Providers’ quality of traumatic shock care score is a validated score calculated by assigning unweighted points for execution of each core component of the bundle of care [23]. Comparisons between the pre- and post-intervention clinical cases were performed on categorical variables using chi square, Mantel-Haenszel chi square, or Fisher’s exact test, as appropriate. Since continuous variables were not normally distributed, comparisons were made using Wilcoxon tests. The statistical software package used for analysis was SAS/STAT version 9.4 (Copyright 2017 SAS Institute, Inc.). Feasibility was interpreted on a range, from favorable to poor, with favorable being the successful calculation of both shock index and quality of care score, and poor being the inability to calculate both, with no realistic solution for the main trial.

The primary feasibility outcome dictated the overall feasibility of the pilot study, while the secondary feasibility outcomes helped qualify whether modifications would be needed for the future main trial.

## Results

**Fig. 2:**
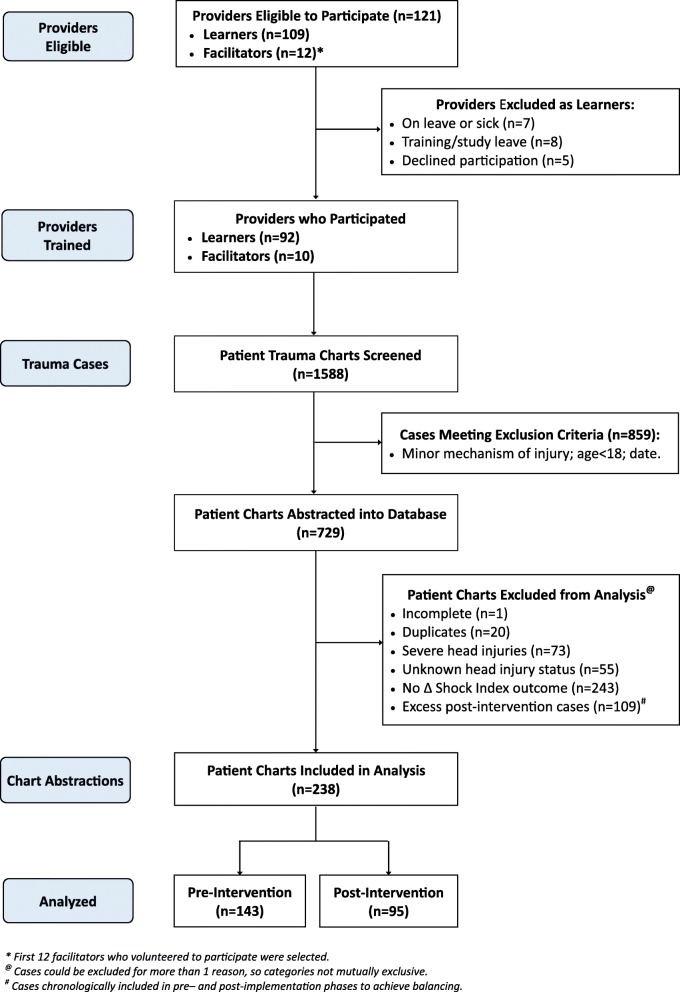
Flow-diagram of provider and trauma patient enrollment

In total, 121 Northern Division providers participated: 12 were trained as facilitators (4 ILS, 8 ALS), and 109 providers (56 BLS, 43 ILS, 10 ALS) were on duty during the implementation period and eligible to participate as learners (Fig. 2). Sixty-five (54%) of all providers were male. The mean age was 37.7 years (standard deviation, SD = 8). All eight EMS-TruShoC modules were delivered by facilitators, resulting in 539 learner encounters and generating 143 h of training time. Learners and facilitators returned completed evaluation forms following 99% and 96% of training sessions, respectively. The average of all implementation indices was 83% (SD = 10.3). All results are summarized in Table 1.

### Reach

Ninety-two of 109 (84%) learners completed at least 6 of 8 (75%) training modules. During interviews, learners explained that training during work time facilitated attendance, and was a major factor for high participation rates (Table 2). Learners contrasted the convenience of on-shift training to traditional CME trainings that occur on their days off, which imposes challenges to their attendance. Facilitators noted learner hesitancy to participate early in the program rollout (Table 3). Reasons for non-participation of 17 learners are in Fig. 2. Lack of advanced notice to learners, and the base managers being hands-off, may have enabled some learners to not participate (Table 4).

**Table 2:** Learner feedback—summarized themes, categorized by topic areas

Topic area	Positive/rewards	Negative/challenges
Content	Relevant to patient care. Important to what we do.	Transport to trauma centers challenging. Scene time < 10 min is challenging.
Format	Enjoy colleagues training us. Enjoy in back of ambulances.	Would like more learners in ambulance. Need opportunity for open discussions.
Timing	Like it at start of (during) shift. Like the short and repeating intervals.	Need more time; 15 min too short. Difficult to train and get on road quickly.
Facilitators	Did a very good job. Brought out all the important points.	Junior medics training senior medics.
Overall	Very happy. Want more. Practical to apply training concepts.	Need more warning of program. Need base management involved.

**Table 3:** Facilitator feedback—summarized themes, categorized by topic areas

Topic area	Positive/rewards	Negative/challenges
On being a facilitator	Enjoyed teaching. Well organized. Initially intimidating, but them comfortable. Elevated my status – I got respect. Educationally useful… for me and learners. Promoted community-building.	Inadequate protected time from operations Initially participants dragging heels and we needed effort to motivate them. Inadequate support from base managers. Teaching was a bit tiring. Catchup weeks challenging.
Facilitators’ training and preparation	Content I imparted was informative. Our training was solid. I was prepared to deliver content.	Had to prepare beyond our trainings. Needed 1–2 more days for our training. Was difficult to get thru material on time.
Comments on training content, materials, and format	Good/informative content. Skills practice was good and enjoyable. Participants enjoyed the trainings Good during shift, no off-days training. Stick figure/diagram of each case was very helpful for learners. Number of modules was okay.	Need a short handout for learners. Need laminated guides for facilitators. Need 5–10 min more for training sessions. 15 min not enough time for Q&A/discussion A bit too much repetition. Evaluation forms and training materials was a lot to juggle.
Supervision from HEET team and base managers	They were generally supportive. HEET Team always checking up on us. The HEET team were easy to interact with. I could ask for help or feedback, if needed.	Need more help working with managers. One manager not very accommodating. Shift managers should be formally part of the program.
HEET compared with traditional training format.	Good not to have to come in on off days. Good to train in short intervals, each shift. Nice to mix knowledge and skills. Cases were very relevant to what we see. Helps me to maintain my national certificate. Good not to sit in class all day.	Wears out the facilitators. Training ALS colleagues is intimidating. ALS does not learn as much as lower ranks. Operational demands a bit distracting.

**Table 4:** HEET team feedback—summarized themes, categorized by topic areas

Topic area	Positive/rewards	Negative/challenges
Priority no. 1. Facilitator: Selection	Broad range of qualifications (ILS & ALS). Facilitators were well-motivated.	Junior ranks training more senior ranks. No advanced advertising to facilitators. Lack of paramedic buy in/support. New employees included as facilitators.
Priority no. 2. Program Ownership	Reasonable collaboration between CQI, HRD, operations, and communications.	Ambulance base management was not formally embedded into the program. No direct oversight by base managers.
Priority no. 3. Facilitator: Training	Covered content well.	Not enough time for training/mastery. Did not “upskill” on facilitation.
Priority no. 4. Program Content and Materials	Content was clinically and locally applicable. Repetition of core concepts was good. Current/evidence-based guidelines. Training was quality-driven from start.	Content was narrow/focused – can add more content as needed (e.g., airway). 1 training material not available (tourniquets). Concern for facilitator burnout.
Priority no. 5. Program Structure: Format	Good for learning. Provides on-going instruction.	TruShoC program felt short for learners. Possibly too many training modules.
Priority no. 6. Program Structure: Logistics	On-shift is good timing. Able to collect all documentation.	Need feedback loop to facilitators. Unsustainable on a large scale?

### Effectiveness

#### Educational outcomes

Pre- vs. post-implementation assessments indicated improvements in trauma resuscitation knowledge, skills, and self-efficacy. Eightfold more learners attained perfect assessment scores for traumatic shock *knowledge* post-training (35 out of 68; 52%) versus pre-training (4 out of 68 = 6%). There was a 3-fold improvement in the proportion of learners achieving perfect performance of traumatic shock *skills* post-training (26 out of 67; 39%) compared with pre-training (8 out of 68; 12%). There was a 2-fold improvement in proportion with high *self-efficacy* in recognizing and managing traumatic shock, assessed by the written questionnaire in the post-training (28 out of 67; 42%) versus pre-training (14 out of 68; 21%) phase. Participants mentioned that the peer-led format, the case-based discussion, and hands-on nature of trainings enhanced knowledge and skills transfer (Tables 2 and 3). Additionally, facilitators reported that the focus on sensitization helped improve learners’ feelings towards trauma care, although there were some items that were out of the control of care providers (e.g., scene time).

#### Clinical outcomes

A total of 1588 clinical trauma cases were screened, of which 739 (47%) met study criteria and were fully abstracted into the study database. Of those cases abstracted, 501 (68%) were excluded for multiple reasons (Fig. 2), with 243 (33%) missing a second set of vital signs, preventing us from calculating delta shock index (∆SI)—in our sensitivity analysis, there was no difference in provider or patient characteristics in the patients excluded versus included in our final analysis. Of the included 238 trauma patients, 143 (60%) received care pre-intervention and 95 (40%) received care post-intervention (Table 5). Seven percent more patients post-training experienced a clinically meaningful improvement in shock index (i.e., absolute decrease in shock index of at least 0.1) compared with pre-training phase (34% versus 27%). Of note, initial shock indices were similar in the pre- versus post-training phases (median (IQR) 0.89 (0.77–1.06) and 0.93 (0.77–1.12), respectively). We observed a clinically significant increase in quality of care in the post-versus pre-training phase (Table 6). Specifically, modest improvements were noted in the following core bundle areas: scene time less than 10 min; delivery of oxygen; transport to a trauma center. There was no clinically meaningful improvement noted in cases with IV catheter placement or controlling external hemorrhage. Overall, 12% of patients received 3 or 4 core bundle items pre-training and 20% received 3 or 4 core bundle items post training. Learners and facilitators explained that case-based and simulation-based training content felt very clinically and contextually relevant, thereby making it easy to readily apply training concepts and skills to clinical care (Tables 2, 3, and 4). We did encounter frequently missing data, particularly in two variables. Of 127 patients with at least 1 IV catheter inserted, no site or IV size was documented in 34 (27%) and 29 (23%) cases, respectively. In 51 cases with hemorrhage control was recorded as being performed, the specific method of control was not documented in 22 (43%) cases.

**Table 5:** Characteristics of pre and post intervention patient populations

Variable	Category	Treatment group	
		Pre-intervention (*N* = 143) % (*N*) or median (IQR)	Post-intervention (*N* = 95) % (*N*) or median (IQR)
Patient demographics			
Patient age		30.0 (23.0–37.0)	29.0 (23.0–37.0)
Patient gender	Female	22% (31)	19% (18)
	Male	78% (112)	81% (77)
Primary injury mechanism	Blunt	42% (60)	44% (42)
	Penetrating	56% (80)	56% (53)
	Other	2% (3)	0% (0)
Vital signs and associated measures			
Systolic blood pressure from initial vital signs		110.0 (95.0–139.0)	100.0 (90.0–130.0)
Heart rate from initial vital signs		106.0 (99.0–114.0)	104.0 (88.0–112.0)
Shock stage from initial vital signs	High (≥ 1.0)	36% (51)	45% (43)
	Intermediate (0.7– < 1.0)	51% (73)	41% (39)
	Normal (<0.7)	13% (19)	14% (13)
EMS response information			
Qualification of provider 1	BLS	25% (35)	24% (23)
	ILS	41% (58)	43% (41)
	ALS	34% (48)	33% (31)
Incident to scene arrival minutes		19.0 (12.0–37.0)	22.0 (14.0–43.0)
Scene arrival to scene departure minutes		22.0 (15.0–32.0)	23.0 (14.0–31.0)
Scene departure to hospital arrival minutes		14.0 (9.0–22.0)	16.0 (12.0–24.0)
Total prehospital minutes		61.0 (48.0–89.0)	67.0 (53.0–96.0)
Clinical interventions			
Any IV is 14,16 or 18-gauge placed in AC or EJ locations	Yes	14% (20)	13% (12)
IV fluids administered:	No	46% (66)	47% (45)
	Yes	50% (71)	41% (39)
	Not documented	4% (6)	12% (11)
Type of fluid:	Normal saline	3% (2)	8% (3)
	Lactated Ringers	96% (68)	90% (35)
	Not documented	1% (1)	3% (1)
Volume of fluid (mL):		825.0 (450.0–1000)	500.0 (250.0–925.0)
Patient shock index outcomes			
Initial shock index		0.89 (0.77–1.06)	0.93 (0.77–1.12)
Last shock index		0.88 (0.74–0.97)	0.87 (0.72–1.00)
Change in shock index		− 0.04 (− 0.12–0.00)	− 0.05 (− 0.17–0.03)
Meaningful change in SI	Meaningfully better (<− 0.1)	27% (38)	34% (32)
	Meaningfully worse (>0.1)	8% (12)	11% (10)
	No meaningful change	65% (93)	56% (53)

*AC* antecubital fossa, *EJ* external jugular, *SI* shock index

**Table 6:** Quality of providers’ shock care

Variable	Category	Treatment group	
		Pre-intervention (*N* = 143) % (*N*)	Post-intervention (*N* = 95) % (*N*)
Scene time category	< 10 min	12% (17)	18% (17)
	10–19 min	29% (41)	23% (22)
	20+ min	59% (85)	59% (55)
Any high flow oxygen treatment	Yes	24% (34)	33% (31)
Any 18G, 16G or 14G IV placed (regardless of location)	Yes	34% (48)	28% (27)
External hemorrhage present and controlled	Yes	13% (18)	14% (13)
Trauma center destination	Yes	31% (45)	34% (32)
Count of core high quality items (range, 0–5)	0	35% (50)	35% (33)
	1	32% (46)	27% (26)
	2	21% (30)	18% (17)
	3	8% (12)	16% (15)
	4	3% (5)	4% (4)
	5	0% (0)	0% (0)
Count of core high quality items (range, 0–5)	0–2	88% (126)	80% (76)
	3–4	12% (17)	20% (19)

### Adoption

Ten of 12 (83%) facilitators actively participated. Two ALS facilitators could not participate in trainings as one went on extended sick leave, and the other was promoted to management. Overall, the participating facilitators were very representative (in gender, rank, and years of experience) compared with the larger pool of eligible facilitators. Qualitative data indicate that, similar to learners, facilitators felt the on-the-job timing, the relative simplicity of training, and enjoyable format, facilitated their high rates of participation. Survey data from key stakeholders’ (managers and HEET Steering Committee) found that an overwhelming majority (92%) support the adoption and incorporation of this program into Western Cape EMS (Table 1). Stakeholders felt HEET was a sound, effective, and sustainable educational solution that aligned well with their EMS organization, and they would adopt the program.

### Implementation Fidelity

The main issue experienced was that 2 modules required implementation in rapid succession during a catch-up phase. Facilitators explained that early in the program, administrative delays and learners “dragging their heels” extended the number of weeks allocated to completing modules 1 and 2. The implementation was adapted by running modules 3 and 4 back-to-back (i.e., “stacked training sessions”), and the intervention was adapted by excluding non-core (airway and breathing management) content to shorten modules 3 and 4. Both adaptations allowed a catch-up phase, and from module 5 onwards, administrators and participants were accustomed with the training regimen, allowing the program to run and conclude on schedule. Overall, 90% of the intervention (i.e., training content) was delivered as intended—about 10% of non-core (“airway” and “breathing”) content was removed from training materials to accelerate the overall program. Overall, 77% of the implementation strategy was delivered as originally planned, calculated by averaging the following three implementation metrics (Table 1): (i) 80% of training sessions achieved the a priori goal of completion in under 20 min, i.e., 15 min for training plus 5 min for logistics; (ii) 77% of sessions started +/− 15 min of shift change, a goal set to minimize disruptions to normal EMS operations; (iii) 75% (6 out of 8) of modules were delivered as planned. Purposeful adaptations were made to approximately 23% of our implementation strategy and 10% of our intervention content [32, 33].

## Discussion

In our single-site pilot study, conducted in a resource-limited, high-trauma, international setting, we found excellent feasibility of both our primary and secondary feasibility endpoints, suggesting we can progress to conduct the main study with existing implementation and data collection strategies. This pilot is the first study to demonstrate that the implementation of EMS-TruShoC using the HEET strategy is highly feasible and implementation and clinical outcomes should be more rigorously evaluated in a larger trial. RE-AIM proved to be an excellent framework to guide the implementation and evaluation of this work. We learned four key lessons from the pilot useful to inform the design, and augment efficient conduct, of the future trial: [1] the on-site training intervention reached a very high percentage of providers and required only minimal to modest training time [2]; some clinical outcomes data were missing with high frequency [3]; collecting clinical effectiveness data consumed a large amount of effort; and [4] the implementation strategy needs to more formally include managers at the ambulance base.

First, the on-site training intervention reached a very large proportion of providers and required only minimal to modest training time (less than 15 min in 80% of trainings). The major contributory factor for high participation was the scheduling of training sessions during work hours, and the inclusion of HEET as part of change of shift procedures. Coupled with the enjoyable training format, learners and facilitators were able to frequently participate. Further, training times were kept short via tight monitoring by EMS shift managers (who released ambulances into service as soon as possible), and by facilitators who were trained to be sensitive to training time.

Second, we found that specific clinical data were missing with high frequency. In our future study, our primary aim is the change in shock index scores [29]. We noted that 33% of cases were missing data necessary to compute this outcome (Fig. 2). Seventeen percent additional cases were excluded for concern for significant head injuries (an a priori exclusion criteria, given different clinical management approaches of head injuries versus hemorrhagic shock). We will need to increase our final sample size by at least 50% in the future study, and tighten our screening criteria, to successfully assess our clinical outcomes. Additionally, we will plan to assess other robust secondary endpoints, including a plan for sensitivity analyses, given missing data.

Third, collecting clinical effectiveness data disproportionately consumed the largest proportion of research effort. We required multiple steps to collect clinical data, starting with a query of the EMS database, followed by manual screening and exclusion procedure, and ending with abstraction into our study database. We excluded 71% of 739 cases originally abstracted (for failing our inclusion criteria, one-third of which were missing our primary variable—change in shock index; Fig. 2)—cumulatively, these processes consumed the most time and personnel effort compared with any other single research activity. This tedious experience predicts that in future research, collecting clinical data will be relatively more complicated and time consuming than collecting implementation or educational data. This experience may explain why most published outcomes of emergency care educational interventions often report proximal (educational) outcomes and fail to assess clinically relevant outcomes [7, 34]. Notwithstanding, we surmounted this challenge and collected all relevant proximal and distal outcomes in this pilot. We may implement strategies to ensure higher screening efficiency for clinical data capture in our future trial.

Last, we repeatedly received feedback, from facilitators and HEET Team, that the challenges experienced implementing HEET (including reluctance by some learners, logistic challenges, and facilitators feeling pressure to go into service) stemmed from slow engagement by shift managers at the base. Shift managers are responsible for ensuring vehicles are staffed and leave the ambulance base on schedule, including other logistic responsibilities. In this pilot, while shift managers were informed about the implementation, they were not formally included in the planning and logistics until implementation. As a result, feedback suggests that while they were supportive of the program, they were disconnected from the details and not adequately invested, resulting in poor oversight and ownership of HEET. The future trial will be modified to formally include managers early and consistently in the implementation process, as recommended in “designing for dissemination” and stakeholder engagement practices [35].

The RE-AIM framework was successfully applied to this work. RE-AIM is a well-known public health framework useful to guide both program and delivery strategy planning and evaluation of implementation outcomes [21, 22]. RE-AIM has been used in a few global health reports from India, Kenya, and Australia, mostly focused on primary care and preventative interventions, we were unable to find published reports of its prior use in prehospital emergency care nor trauma care internationally [22]. Hence, we have demonstrated a proof of use of RE-AIM in a new context, which helps fill a gap in implementation science. Overall, we found RE-AIM easy to apply given its inherent simplicity and practicality as an implementation science framework [36]. We anticipate RE-AIM will offer similar ease of application in our future, planned large-scale study. Of note, we do plan to assess the fifth RE-AIM dimension (maintenance) in our future study that will have a longer follow-up period.

Our experience from this pilot study demonstrate three important concepts: first, that EMS-TruShoC can be practically implemented with HEET, and evaluated, in a busy EMS base in Africa; second, that relevant clinical outcomes data (shock index and quality of care scores) EMS-TruShoC are highly feasible to collect; and third, that there is a modest clinical outcomes benefit that warrants further assessment. We received generally positive and enthusiastic feedback from participants and stakeholders about the intervention and implementation strategy. Our pilot experience holds exciting promise for future implementation and research efforts.

## Limitations and strengths

Study strengths included the multi-method (qualitative and quantitative) approach, the systematic assessment of important implementation issues using the RE-AIM framework, and inclusion of both learner and clinical patient outcomes. There are a few limitations of this pilot study. First, the study was conducted in only one site and we did not formally assess organizational context at the Northern Division base—while the future study will be conducted in the same EMS organization but with multiple sites, defining and understanding local context may be important for replication. Second, we did not assess cost-effectiveness—while we have a gross estimation of the resources and inputs, a formal micro-costing analysis may help predict costs of the future trial [37]. Third, we did not assess maintenance of the intervention in this pilot—this program is deliberately designed to have sustainable effectiveness, and although we do not foresee challenges to maintenance in our future trial, we cannot be certain as maintenance was not measured.

## Conclusions

In this single-site pilot study, we successfully implemented the EMS-TruShoC educational intervention using the HEET training strategy, in a low-resource international setting. We successfully collected and analyzed quantitative and qualitative data on implementation, educational, and clinical effectiveness that will help enhance the conduct of our future trial. Overall, this pilot study suggests excellent feasibility and value of our future, planned experimental trial.

## Data Availability

The datasets used and/or analyzed during the current study are available from the corresponding author on reasonable request.

## Abbreviations

EMS: Emergency medical services
HEET: High-efficiency EMS training
RE-AIM: Reach, efficacy, adoption, implementation fidelity, maintenance
TruShoC: Traumatic shock care
